# O12 - Specific IgE levels for goat’s and sheep’s milk after successful oral immunotherapy to cow’s milk in children

**DOI:** 10.1186/2045-7022-4-S1-O12

**Published:** 2014-03-14

**Authors:** Liseth Villafana, Soledad Terrados, Belen De la Hoz, Mónica Rodriguez, Ana De Andrés

**Affiliations:** 1 Ramón y Cajal Hospital, Madrid, Spain; 2 San Carlos Clinic Hospital, Madrid, Spain

## Introduction

Cow’s milk (CM) oral immunotherapy has proven to be effective as a treatment for persistent CM allergy. Allergic reactions to goat and sheep cheeses have been described in almost 25% of patients desensitised to CM, indicates that immunotherapy is specific for each milk.

## Objectives

To describe changes of goat’s IgE and sheep’s IgE in patients who tolerated CM after a CM oral immunotherapy.

## Methods

24 children aged between 4 and 13 who achieved tolerance to milk after CM immunotherapy were included. Specific IgE to cow’s caseine, sIgE to whole goat’s milk and sIgE to whole sheep’s milk at baseline and at 24 months follow up were analised (CAP-Phadia). The patientes were classified in two groups: high risk (17 patients) and low risk (7 patients) based on the premedication need, number of adverse reactions and time to achieve a complete tolerance to CM.

## Results

The median of Specific IgE to CM casein, goat’s whole milk, sheep’s whole milk a baseline were 24.3KU/L, 31.2KU/L and 27.4KU/L and 9.5KU/L, 16.9KU/L and 17.3KU/L after 24 months of follow up (p<o,o5).In the high risk patients the median of Specific IgE to CM casein, goat’s whole milk, sheep’s whole milk were 33.7KU/L, 43.7KU/L and 37.1KU/L at baseline and 13.1KU/L, 23.9KU/L and 23.6KU/L after 24 of follow up (p<o,o5). In contrast, in the low risk group the specific IgE to CM casein, goat’s whole milk, sheep’s whole milk did not shown a significant decrease after 24 of follow up. 4 patients showed a different patron referred to IgE-goat’s milk and IgE-sheep’s milk compared with CM, independently of risk group.

## Conclusions

We found a decrease of IgE levels to whole goat´s and sheep´s milk after oral immunotherapy to CM.

